# Development of New Genome Editing Tools for the Treatment of Hyperlipidemia

**DOI:** 10.3390/cells12202466

**Published:** 2023-10-16

**Authors:** Giulio Preta

**Affiliations:** 1 VU LSC-EMBL Partnership Institute for Genome Editing Technologies, Life Sciences Center, Vilnius University, LT-10257 Vilnius, Lithuania; giulio.preta@bchi.vu.lt; 2 Institute of Biochemistry, Life Science Center, Vilnius University, LT-10257 Vilnius, Lithuania

**Keywords:** CRISPR-Cas9, base editing, cardiovascular disease, LDL cholesterol, PCSK9

## Abstract

Hyperlipidemia is a medical condition characterized by high levels of lipids in the blood. It is often associated with an increased risk of cardiovascular diseases such as heart attacks and strokes. Traditional treatment approaches for hyperlipidemia involve lifestyle modifications, dietary changes, and the use of medications like statins. Recent advancements in genome editing technologies, including CRISPR-Cas9, have opened up new possibilities for the treatment of this condition. This review provides a general overview of the main target genes involved in lipid metabolism and highlights the progress made during recent years towards the development of new treatments for dyslipidemia.

## 1. Introduction

Genome editing refers to the modification of an organism’s DNA to alter its genetic information. One of the most promising genome editing tools is CRISPR-Cas9, which stands for clustered regularly interspaced short palindromic repeats (CRISPR) and CRISPR-associated Protein 9 (Cas-9). CRISPR-Cas9 allows scientists to make precise changes in the DNA sequence by targeting specific genes and introducing modifications [1,2]. In the context of hypercholesterolemia, the main goal is to target genes involved in cholesterol metabolism to reduce the levels of low-density lipoprotein (LDL) cholesterol in the blood. Since the liver plays a critical role in the production and clearance of lipoprotein particles, genome editing strategies are optimized to target genes within hepatocytes. For instance, adeno-associated virus (AAV) vectors based on serotype 8 have a specific tropism for the liver and have been used in several studies for somatic genome editing in mice [3,4]. Lipid nanoparticles (LNP) are also delivery vehicles for CRISPR-Cas9 editing and are efficiently taken up by hepatocytes due to their ability to interact with serum proteins [5].

Researchers have been exploring the use of genome editing to develop novel therapies as an alternative to the existing ones including statins, ezetimibe, PCSK9 (proprotein convertase subtilisin/kexin type 9) inhibitors, niacin, bile acid sequestrants, fibrates and bempedoic acid [6,7,8,9]. Statins were first introduced for the treatment of high cholesterol levels in the late 1980s. The first statin to be approved for clinical use was lovastatin (Mevacor) in 1987. Following the approval of lovastatin, other statins were subsequently developed and introduced for the treatment of hypercholesterolemia. Some of the commonly prescribed statins include simvastatin (Zocor), atorvastatin (Lipitor), pravastatin (Pravachol), and rosuvastatin (Crestor). While statins are considered safe and effective, there are different observed side effects associated with their use, including muscle pain and weakness, gastrointestinal symptoms, and liver enzyme abnormalities, which led to the development of alternative or complementary therapies [10,11]. Ezetimibe is often considered a valid option for individuals who cannot tolerate statins or require additional LDL reduction [12]. This medication acts by reducing the absorption of cholesterol from the intestine, selectively inhibiting the protein Niemann-Pick C1-Like 1 (NPC1L1), which is responsible for transporting cholesterol from the intestine into the bloodstream [13]. In clinical studies, when used as a monotherapy, ezetimibe was able to reduce LDL cholesterol by 18% [14], while when used in combination with statins, it provides a variable reduction according to the statin used, sample size, and dosage [15,16].

PCSK9 inhibitors (evolocumab and alirocumab) are monoclonal antibodies that specifically target PCSK9 [17,18]. When injected, these antibodies bind to circulating PCSK9 molecules, preventing them from interacting with LDL receptors (LDLRs). By blocking the interaction between PCSK9 and LDLRs, PCSK9 inhibitors hamper the internalization of LDLRs, allowing them to remain on the surface of cells and increasing the liver’s capacity to capture LDL particles from the bloodstream [19,20]. However, there are still several obstacles to the clinical use of PCSK9 inhibitors including the appearance of several side effects and the cost of its clinical use in relation to its effectiveness [21]. The use of small interfering RNA (siRNA) represents another strategy to inhibit the internalization of LDLRs. Inclisiran specifically targets the 3` UTR of PCSK9 mRNA, entering hepatocytes through asialoglycoprotein receptors and leading to an increased expression of LDLR receptors in the membranes [22]. The results on its efficacy are based mainly on three clinical trials named ORION-9, ORION-10, and ORION-11 showing a decrease in LDL-C, lipoprotein(a) [Lp(a)], and triglycerides (TG). All ORION clinical trials are reviewed by Katsiki and colleagues [23]. The long-term safety and efficacy of inclisiran will be evaluated in the ongoing trial ORION-4, which will determine the clinical relevance of this promising new treatment [8]. Evinacumab is another monoclonal antibody that pharmacologically inhibits angiopoietin-like 3 (ANGPTL3) and was recently approved by the FDA as a complementary agent to other LDL-C-lowering therapies for patients with homozygous familial hypercholesterolemia (HoFH). The binding of evinacumab to ANGPTL3 preserves the function of lipoproteins and endothelial lipase, leading to a decrease in total cholesterol (TC), TG, and LDL-C [24]. Similar results were observed with the apolipoprotein C3 (ApoC3) inhibitor olezarsen, a hepatocyte-targeted, GalNAc-modified antisense oligonucleotide that decreases the plasma levels of ApoC3 and consequently reduces triglycerides levels in subjects with high cardiovascular risk [25,26]. Pelacarsen is an antisense oligonucleotide covalently bonded to GalNAc, which prevents the production of apoliporotein(a) [Apo(a)]. Apo(a) is encoded by the *LPA* gene and should not be confused with members of the apolipoprotein A family, encoded by different genes (i.e., *APOA1*, *APOA2*). The binding of Apo(a) to ApoB100 on LDL leads to the formation of Lp(a). Phase 1 and phase 2 pelacarsen clinical trials showed a considerable decrease in the serum level of Lp(a) [27,28]. Olpasiran is a siRNA that blocks the assembly of Lp(a) by inhibiting the translation of Apo(a) in the hepatocytes. Several clinical trials proved its efficiency and safety, promoting the development of additional siRNA in order to reduce Lp(a) blood levels [29,30,31].

Niacin was considered a powerful drug for the treatment of lipid abnormalities, acting by decreasing fatty acid mobilization from adipose tissue and by inhibiting triglyceride synthesis [32]. However, two large randomized clinical studies have recently shown disappointing results, leading to the conclusion that there are no effective benefits to adding niacin to existing statins therapy for patients with high cardiovascular risk [33,34]. Limitations in the design of these two clinical trials as well as the possibilities for usage of niacin for specific types of dyslipidemias are described in a study by Zeman and colleagues [35]. Bile acid sequestrants such as cholestyramine, cholestipol, or colesevelam, due to their high level of charged molecules, bind to negatively charged bile acids in the intestine, inhibiting cholesterol absorption. A growing amount of evidence suggests that they play a role not only in lipid but also in glucose metabolism [36,37]. Bile acid sequestrants can be used as monotherapy or in combination with statins or ezetimibe. Moreover, since they are not absorbed in the gastrointestinal tract, they have limited toxicity [38].

**Figure 1:**
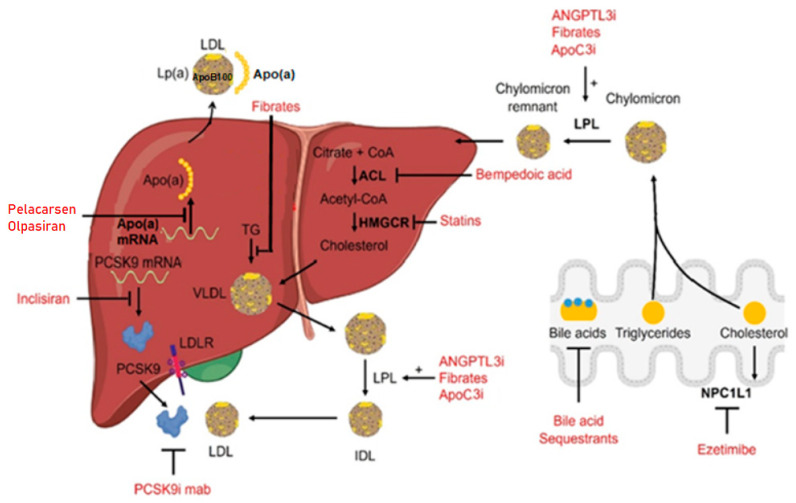
Main therapies used for the treatment of hyperlipidemia with the associated mechanism of action. Different drugs are indicated in red, and the arrows point to the specific molecular targets. A few compounds can act at different levels. Figure modified from [9], licensed under CC-BY 4.0.

Treatment with fibrates results in a substantial decrease in plasma triglycerides and is also associated with a slight reduction in LDL cholesterol [39,40]. The effects of fibrates are related to alterations in the transcription of genes encoding for proteins that control lipoprotein metabolism. More specifically, the primary target of fibrates is the peroxisome-proliferator-activated receptor alpha (PPAR-alpha). The binding of fibrates to PPAR-alpha receptors induces their activation and the formation of a complex between PPAR-alpha and the retinoid X receptor (RXR). This PPAR-alpha/RXR complex binds to specific DNA sequences known as peroxisome proliferator response elements (PPREs) in the promoter regions of target genes, leading to the upregulation of genes involved in lipid metabolism, especially those responsible for fatty acid oxidation in the liver and muscles [41,42]. Bempedoic acid is a novel LDL-cholesterol-lowering agent, inhibiting adenosine triphosphate citrate lyase, an enzyme involved in the cholesterol synthesis pathway, upstream from the HMG-CoA reductase [12]. Several clinical trials have demonstrated the efficiency of this drug when used as a monotherapy or in combination with other lipid-lowering therapies [43,44,45]. In Figure 1, a schematic outline of the above-described drugs’ mechanisms for lowering serum lipids is presented.

## 2. *PCSK9* Gene Editing Strategies in Mice

The *PCSK9* gene plays a role in regulating cholesterol levels by controlling the number of LDL receptors on the surface of liver cells. To become fully functional, PCSK9 protein undergoes a maturation process involving several steps: PCSK9 is synthesized in the endoplasmic reticulum (ER) in the form of inactive zymogen, called PreProPCSK9. PreProPCSK9 is composed of five parts: a signal peptide, the pro-domain, the catalytic domain, the hinge region, and the C-terminal domain. The protein is then subjected to autocatalytic cleavage in the ER in order to lose its signal peptide, and it becomes ProPCSK9. ProPCSK9 is then transported to the trans-Golgi network (TGN), where it undergoes proteolysis to form mature PCSK9 [46,47]. Only the mature form is then transported in endosomes and secreted into the circulation, where it binds to the LDL receptor on the surface of hepatocytes. The PCSK9-LDLR complex is internalized into the cell via endocytosis. Inside the cell, the LDLR is targeted for lysosomal degradation instead of being recycled to the cell surface for further use. In this way, with fewer functional LDL receptors available on the cell surface, the liver becomes less efficient at clearing LDL cholesterol from the bloodstream [48]. This leads to higher levels of LDL cholesterol in the blood and to the potential risk of cardiovascular diseases. Autosomal dominant variants of the *PCSK9* gene can lead to a condition called familial hypercholesterolemia (FH), which is characterized by high levels of LDL cholesterol and an increased risk of cardiovascular disease [49,50]. *PCSK9* genome editing strategies aim at loss-of-functions mutations that are always associated with reduced plasma levels of LDL-C in nature [51,52,53,54]. Researchers have used CRISPR-Cas9 to disrupt or modify the *PCSK9* gene, effectively reducing the production of PCSK9 protein and the levels of LDL cholesterol [55,56]. In the first in vivo study in mice, conducted by Ding and colleagues, the authors selected a gRNA-targeting exon 1 of mouse *PCSK9* and generated an adenovirus expressing this gRNA and Cas9. As soon as 3 to 4 days after injection of the adenovirus, P mutagenesis had occurred in more than half the mice, resulting in decreased PCSK9 levels and reductions in TC of up to 40%. No off-target effects were observed [57]. A few years later, in a mouse model with humanized hepatocytes, adenovirus was used again as a vector to deliver gRNA-targeting exon 1 of *PCSK9*. After a few days, almost 50% mutagenesis was observed, with a reduction in human levels of PCSK9 of 52%. Interestingly, mouse PCSK9 protein levels increased, probably as a compensatory mechanism, and no effect on TC was observed [58]. The limitation of these two studies was related to the use of adenovirus as a vector, since their long persistence and immunogenicity in the host prevent the potential therapeutic applications in humans [59,60]. AAV-mediated delivery of the CRISPR-Cas9 system has shown high gene targeting efficacy in vivo and a lower immunogenicity emerging as an alternative delivery method for the CRISPR-Cas9 system to various cell types, tissues, and organs [61,62]. Ran and colleagues used an adeno-associated virus and a Cas9 orthologue from *Staphylococcus aureus* (for its smaller size) instead of the *Streptococcus pyogenes* Cas9. The mutagenesis observed in *PCSK9* gene was greater than 40%, the reduction in PCSK9 levels reached 95%, and the reduction in cholesterol levels was 40% [63]. Since long-term expression of Cas9 in target cells creates concerns related to toxicity and appearance of off-target effects, a self-cleaving AAV-CRISPR-Cas9 system was developed. This system can effectively eliminate Cas9 protein expression without compromising the editing efficacy of the *PCSK9* gene and reducing the off-target effects [64]. A completely different approach was used in a 2017 study, where newly developed lipid-like nanoparticles (LLNs) were used successfully to deliver Cas9 RNA- and gRNA-targeting *PCSK9* in the livers of mice [65,66]. The targeting was effective for both episomal and chromosomal DNA, and since Cas9 mRna/protein and gRNA were degraded within one day in mice, this methodology provides a temporarily controllable way to achieve in vivo genome editing. A strategy termed selective organ targeting (SORT) was developed to allow lipid nanoparticles to be engineered for precise delivery of different cargoes including mRNA, Cas9 mRNA/single-guide RNA (sgRNA), and Cas9 ribonucleoprotein (RNP). When this methodology was used for *PCSK9* targeting, a significant indel induction at the *PCSK9* locus (~60%), corresponding to a ~100% reduction in PCSK9 levels in the liver and blood, was observed [67]. Lipid fats were also used as a delivery method in a study where chemically modified gRNA was developed. These modifications did not inhibit the interaction between gRNA and Cas9, while maintaining or even enhancing the genome editing activity, leading to a >80% editing of in the liver with a single injection [68].

To address the lack of effective models to test the efficacy of techniques to target human *PCSK9*, Carreras and colleagues developed a liver-specific human *PCSK9* knockin mouse model (hPCSK9-KI) [69]. Human *PCSK9* was expressed in the liver from hPCSK9-KI but not from their wild-type littermates, whereas the expression of endogenous mouse PCSK9 mRNA was comparable in the liver between the two types of mice. In this model, CRISPR-Cas9-mediated genome editing of human *PCSK9* decreased plasma levels of human but not mouse PCSK9, and in parallel, it reduced the plasma concentrations of cholesterol, while genome editing of mouse *PCSK9* did not affect cholesterol levels. Base editing using a guide RNA that targeted both human and mouse *PCSK9* reduced the plasma levels of human and mouse PCSK9 and cholesterol levels. Therefore, this model can be used for the evaluation of genome-/base-editing therapies to regulate the expression of PCSK9 and consequently the blood cholesterol levels.

## 3. *PCSK9* Gene Editing Strategies in Non-Human Primates

The first in vivo gene editing study in non-human primates (NHPs) for PCSK9 was carried out using an AAV vector expressing an engineered meganuclease targeting *PCSK9* [70]. Meganucleases have been more challenging to engineer for new target sequences, making them less versatile than CRISPR-Cas9; however, advances in protein engineering have improved their flexibility [71,72]. The meganucleases were used in Rhesus macaques, and a reduction in PCSK9 levels of up to 84% was observed, while LDL-C reduction was up to 60%. Several off-target effects were registered during the study, as well as the induction of an immune response [70]. To overcome these unwanted effects, related to the use of an AAV vector, lipid nanoparticles were used as a delivery system [73]. CRISPR base editors, delivered in vivo using lipid nanoparticles, can efficiently target the *PCSK9* gene in *Macaca fascicularis*. A near-complete knockdown of *PCSK9* in the liver after a single infusion of lipid nanoparticles was achieved. This led to a reduction in blood levels of PCSK9 and low-density lipoprotein cholesterol of approximately 90% and 60%, respectively. Compared with Wang and colleagues’ study, no significant off-target activity was observed. Lipid nanoparticles were also used for the delivery of mRNA encoding an adenine base editor (ABE) and a single-guide RNA targeting *PCSK9* [74]. ABEs consist of a catalytically impaired Cas9 protein fused with an adenine deaminase and a modified gRNA. The modified gRNA guides the ABE to the target DNA sequence. The adenine deaminase enzyme then chemically modifies the adenine base in the DNA to become inosine, which is recognized as guanine by cellular machinery. During DNA replication, the complementary cytosine is added to the modified adenine, leading to a G•C base pair conversion [75]. *PCSK9* base-editing in Cynomolgus monkeys occurred in a mean of 26%, while the reduction in PCSK9 protein was 32% and 14% for LDL-C. In this study as well, no off-target editing was observed. VERVE-101, an investigational CRISPR base editing therapy, consists of an mRNA for an adenine base editor and a gRNA targeting the *PCSK9* gene assembled in a lipid nanoparticle delivery system [76]. Liver biopsies 14 days after Cynomolgus monkeys were given a single intravenous infusion of a vehicle control or VERVE-101 at a dose of 0.75 mg/kg or 1.5 mg/kg showed *PCSK9* editing of 46% (0.75 mg/kg) and 70% (1.5 mg/kg). The related reduction in low-density lipoprotein cholesterol was 49% (0.75 mg/kg) and 69% (1.5 mg/kg). These promising results led Verve Therapeutics to start human clinical trials using VERVE-101 in patients with FH [77].

## 4. *ANGPTL3* Gene Editing Strategies in Mice

**Figure 2:**
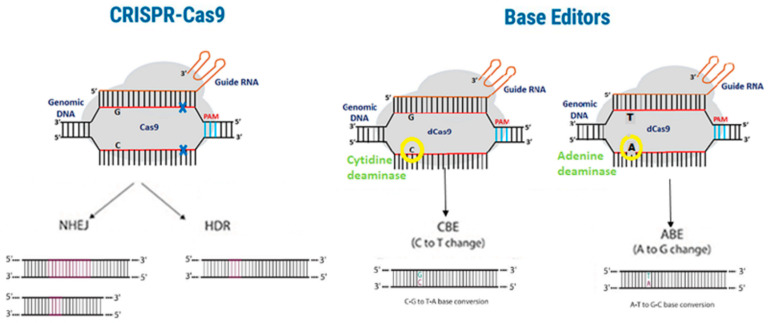
Differences between the CRISPR-Cas9 and base editors methodologies. Traditional CRISPR-Cas9 gene editing (left panel) introduces double-strand breaks, which can lead to off-target effects. Base editing (right panel) avoids double-strand breaks due to catalytically inactive Cas9 (dCas9), thereby limiting the occurrence of off-target effects.

*ANGPTL3* is a gene that produces a protein involved in lipid metabolism. ANGPTL3 plays an important role in regulating triglycerides and cholesterol blood levels via the inhibition of lipoprotein lipase and endothelial lipase enzymes activity [78,79]. Loss-of-function mutations in this gene have been associated with lower LDL cholesterol levels and a reduced risk of cardiovascular diseases [80,81]. The potential benefits of targeting *ANGPTL3* were confirmed in a study, using antisense oligonucleotide. An effective reduction in ANGLPT3 protein levels was achieved, and the same was achieved for TG and LDL cholesterol [82]. Intravenous injection of a specific monoclonal antibody in dyslipidemic C57BL/6 mice also reduced TG, LDL-C, and HDL-C levels in the blood [83]. Scientists have recently explored the use of CRISPR-Cas9 to disrupt or modify the *ANGPTL3* gene to mimic the effects of these beneficial mutations and reduce LDL cholesterol levels. The base-editing approach was tested, which allows us to alter specific nucleotides in the DNA sequence without generating double-strand breaks (Figure 2) [84]. The authors produced an adenoviral vector expressing base editor 3 targeting *ANGPTL3* and injected this vector into C57BL/6J mice. This resulted in reduced plasma ANGPTL3, triglyceride, and TC levels (49%, 31%, and 19%, respectively) [85]. The effect was even bigger when hyperlipidemic *LDLR* knockout mice were injected (triglycerides reduction by 56% and cholesterol reduction by 51%). Interestingly, this study also compared the effects of targeting *ANGPLT3* versus *PCSK9*. *ANGPTL3*-targeted therapy is a more potent triglycerides-lowering therapy, whereas *PCSK9*-targeted therapy is a more potent LDL-lowering therapy. Moreover, inhibiting both ANGPTL3 and PCSK9 did not result in any synergistic or additive effects. Lipid nanoparticles were also used for the delivery of Cas9 mRNA and gRNA for CRISPR-Cas9-based genome editing of *ANGPLT3* in mice. This delivery system has reduced delivery efficiency compared to viral vectors, but possesses less undesired insertional mutagenesis and potential biosafety issues [86,87]. Liver-specific knockdown of *ANGPTL3* resulted in a profound lowering of LDL-C and triglycerides levels. No evidence of off-target mutagenesis was detected, nor of any liver toxicity, and the genome editing retains a therapeutically relevant level for at least 100 days after the injection of a single dose [88]. In another study, where base editing of *ANGPTL3* via AAV delivery was used in C57BL/6J mice, the authors managed to achieve a near-complete knockout of the ANGPTL3 protein in the circulation and a reduction in serum levels of triglyceride and TC by 58% and 61%, respectively [89]. Evaluation of liver toxicity was also conducted, showing no significant changes in the levels of aspartate aminotransferase (AST) and alanine aminotransferase (ALT) and no T cells infiltration or general sign of inflammation. While the above-mentioned study uses a dual-AAV base editor, Davis and colleagues developed a single-AAV adenine base editor system that supports robust editing in vivo and has a broad targeting capability [90]. The use of a single AAV vector for delivery guarantees a maximum editing efficiency, making it the best option when targeting non-liver tissues, or when toxicity limits AAV dosage. In mice, single-AAV-encoded ABE led to a knockdown of both PCSK9 and ANGPTL3 > 90% and to a reduction in circulating cholesterol.

## 5. *ANGPTL3* Gene Editing Strategies in Non-Human Primates

An alternative approach to the use of lipid nanoparticles as a delivery system is a multi-valent *N*-acetylgalactosamine (GalNAc)-targeting ligand, which allows for uptake via the asialoglycoprotein receptor (ASGPR) pathway [91]. Delivery via ASGPR has several positive aspects: the receptor is highly expressed in the liver but not in other tissues of the body, induces an immediate endocytosis of the drug candidate when bound by GalNAc, and is rapidly recycled to the hepatocyte surface [92]. This methodology was selected for CRISPR base editing therapy targeting the *ANGPTL3* gene in NHPs. A mean liver *ANGPTL3* editing of 61% was observed in the six LDLR-deficient NHPs treated with the GalNAc-LNPs, corresponding to a reduction in blood ANGPTL3 protein of 89%. Circulating LDL-C also fell by 35%, a stable reduction for three months after treatment [93]. Liver toxicity tests registered only a transient increase in ALT and AST. In WT NHPs, a mean reduction in blood ANGPTL3 protein of 90% was noted for animals treated with the GalNAc-LNP versus 75% in those treated with a standard LNP. However, WT NHPs showed minimal changes in LDL-C despite a significant ANGPTL3 reduction. This is in line with prior preclinical data on NHPs of a monoclonal antibody targeting ANGPTL3 [83]. Based on this study, currently, VERVE Therapeutics is conducting a trial with its candidate VERVE-201 in a larger 34 NHPs sample.

## 6. *LDLR* Gene Editing Strategies in Mice

The *LDLR* gene encodes a receptor that plays a crucial role in regulating cholesterol levels in the body by allowing cells to take up cholesterol-rich LDL particles from the blood. Several cases of FH are related to mutations of the *LDLR* gene [94,95]. Mutations in *LDLR* can impair LDLR activity at different levels and are classified according to their phenotypic behavior as class 1 (no protein synthesis), class 2 (partial or complete retention of LDLR in the endoplasmic reticulum), class 3 (defective binding to apolipoprotein B), class 4 (defective endocytosis), and class 5 (decreased LDLR turnover ability) [96]. The role of the *LDLR* gene in lipid metabolism was also investigated using CRISPR-Cas9. AAV-CRISPR-Cas9 was used to disrupt the hepatic *LDLR* gene in adult mice, leading to severe hypercholesterolemia and atherosclerotic lesion in the aortas of C57BL/6J mice [97]. Similar observations were made in another study, where an *LDLR^E208X^* mutant knockin mouse model was generated. This model is based on an E207X nonsense point mutation in *LDLR*, observed in individuals with FH, and led to severe atherosclerosis as a consequence of the total depletion of LDLR expression [98]. However, when the mutant *LDLR^E208X^* strain was treated with AAV-CRISPR-Cas9, LDLR expression was partially restored, and the signs of atherosclerosis were mitigated, highlighting the potential use of CRISPR-Cas9 in the treatment of the HoFH [99]. Greig and colleagues used *LDLR*^−/−^ mouse to test their AAV8 vectors expressing both murine and human versions of LDLR [100]. These vectors were previously used in double knockout mouse models, resulting in a complete correction of hypercholesterolemia [101,102]. Minimal levels of toxicity and inflammation response (cytokines production) were observed in the study, while a stable reduction in cholesterol was achieved with the lowest doses of LDLR vectors.

## 7. *LDLR* Gene Editing Strategies in Other Animal Models

Rabbits and hamsters have been widely used as animal models in the study of atherosclerosis because they have similar lipoprotein metabolism to humans and are more susceptible to atherosclerosis [103,104]. *LDLR*-KO rabbits with biallelic mutations were created to induce spontaneous hypercholesterolemia and atherosclerosis on a normal chow diet. Analysis of their plasma lipids showed an increase in triglycerides and a parallel decrease in HDL-C [105]. *LDLR*-KO hamsters can be induced by microinjecting CRISPR-Cas9 components into fertilized eggs for the development of hypercholesterolemia and hyperlipidemia models [106]. In line with the FH patients with *LDLR* gene mutations who have severe hypercholesterolemia in their homozygous form and a moderate hypercholesterolemia in the heterozygous form, *LDLR* −/− hamsters exhibit a severe form of hypercholesterolemia, while *LDLR* +/− hamsters exhibit a moderate form. This behavior differs compared with other species, including mice, where in the heterozygous form, there is never a significant increase in cholesterol levels, making hamsters an optimal tool for research on human atherosclerosis [107,108].

## 8. Apolipoproteins Gene Editing Strategies in Mice

Apolipoproteins are protein components associated with lipoproteins that have several functions, including stabilizing the structure of lipoproteins, serving as ligands for cellular receptors, and participating in enzymatic reactions [109]. Apolipoproteins can be classified into two subgroups: the soluble apolipoproteins including ApoA1, A2, A4, C1, C2, C3, and E, and the insoluble forms like ApoB100 and ApoB48. Alterations in their expression levels or spatial structure are closely related to a variety of diseases [110]. ApoA1 is the primary structural component of HDL particles and is a key mediator of plasma cholesterol transport and cholesterol homeostasis, interacting with several transporters and receptors [111,112]. De Giorgi and colleagues targeted the *APOA1* locus with AAV delivery of CRISPR-Cas9 in mice and achieved rates between 6% and 16% of targeted hepatocytes, with no evidence of toxicity. In this study, improved expression of transgenic proteins from the *APOA1* locus enhanced the expression of ApoE, reducing plasma lipids in a model of hypercholesterolemia [113]. The *APOB* gene provides instructions for the production of apolipoprotein B, a protein that is essential for the assembly and transport of LDL cholesterol in the bloodstream. Mutations in the *APOB* gene can cause familial hypercholesterolemia, a genetic disorder characterized by extremely high LDL cholesterol levels [114]. Mice treated with AAV-CRISPR vectors to disrupt the *APOB* gene showed a significant decrease in plasma cholesterol and were protected from atherosclerosis [3]. However, the treatment exacerbated hepatic fat accumulation, resulting in a microvesicular steatosis, as observed in humans with naturally occurring loss-of-function mutations in *APOB* [115]. Fat accumulation was also observed in another study where the *APOB* gene was targeted, using hepatocyte-tropic AAV-8 serotype [63].

## 9. Apolipoproteins Gene Editing Strategies in Other Animal Models

ApoC3 is another key regulator of plasma triglycerides and is found on chylomicrons, VLDL, LDL, and HDL particles. Recent studies have shown that ApoC3 levels are an independent risk factor for cardiovascular diseases (CVD) [116,117]. Despite the overexpression of human ApoC3 significantly accelerated atherosclerotic development in mice, the protective effect of ApoC3 deficiency on atherogenesis was not observed in KO mice [118]. However, inactivation of the *APOC3* gene by CRISPR-Cas9 in hamsters, led to a decrease in plasma tryglicerides and to an enhanced conversion of VLDL into LDL. When the hamsters were fed with a high-cholesterol diet, a clear reduction in atherosclerotic lesions was observed [119]. *APOC3* KO rabbits were also generated and displayed triglyceride levels 50% lower than those of the age-matched control group when given a normal chow diet. When fed with a high-fat diet, the *APOC3* KO rabbits showed limited atherosclerotic lesions, while the WT rabbits had obvious atherosclerotic lesions, as well as increased intima thickening, collagen content, and levels of inflammatory cytokines (IL-1β and TNF-α) [120]. The limitations of this study were the usage of only three *APOC3* KO rabbits without a consistent genotype, suggesting that further in vivo studies are required to elucidate the impact of *APOC3* knockout on hyperlipidemia. A summary of animal model studies targeting genes associated with dyslipidemia is presented in Table 1.

## 10. Off-Target Effects in CRISPR-Cas9 Gene Editing

A major concern in the application of CRISPR-Cas9 gene editing technologies is the occurrence of off-target effects. Off-target effects are defined as unintended cleavage and mutations at untargeted genomic sites showing similarity with the target sequence [121]. Several studies have shown that Cas9 binds to unintended genomic sites and creates double-strand breaks, leading to undesired outcomes [122,123]. These off-target sites are often gRNA-dependent, since Cas9 is known to tolerate up to three mismatches between the gRNA and the genomic DNA [124]. However, recent findings suggest that gRNA-independent off-target effects could also occur with base editors as a consequence of random deamination [125,126]. Off-targeting can cause severe problems for the host organism, since it could lead to chromosomal rearrangements, loss of functional gene activity, or activation of oncogenes [127,128]. It is therefore crucial for the future therapeutic use of CRISPR-Cas9 gene editing to limit the occurrence of off-target effects by designing a well-engineered CRISPR system with high on-targeting efficiency. There are several strategies that are currently used to achieve this goal including the increase in nucleases cleavage specificity. In recent years, several new Cas9 proteins have been developed, such as Sniper-Cas9 and HypaCas9 [129,130]. Alternatively, a mutated form of Cas9 acts as a nickase (nCas9), where one of the endonuclease domains is catalytically inactivated. This leads to a cut in just one of the two DNA strands, creating a single-strand break. A second nCas9 targeting the opposite strand completes the double-strand break. This variant of Cas9 is able to reduce off-target effects by up to 1500 times compared with its wild-type form, but it is limited by the need for two appropriately spaced gRNAs acting on opposite strands [124,131]. Another strategy is to modify the gRNA: several studies show that the specificity of Cas9 activity can be increased by extending or truncating gRNA [132,133]. Additional decreases in unwanted mutagenesis are observed when the modified gRNA is associated with Cas9 nickase [134]. The selection of an appropriate delivery method for Cas9/gRNA is crucial not only for inducing low immunogenicity in the host, but also because it can profoundly affect the occurrence of off-target effects [135]. AAV-based gene delivery is known to last for years in terminally differentiated cells or exhibits a higher tendency to induce unwanted off-target effects over time [136,137] to trigger an immune response [138]. In contrast, LNP-delivered Cas9 are rapidly degraded in vivo, decreasing the opportunity for off-target activity during in vivo genome editing [88,139]. Choosing an appropriate off-target detection method such as biased and unbiased methods with predictive on-target and off-target sites is a necessary step in preventing the occurrence of undesired mutagenesis. Differences among the several off-target detection methods are outside the scope of this manuscript, but several reviews extensively describe the available tools [140,141].

## 11. Conclusions

Hyperlipidemia is definitely a suitable condition to address with genome editing, since preclinical studies have demonstrated that the targeting of genes associated with cholesterol levels is feasible. Currently, there are already several attractive targets for liver-directed genome editing that could lower lipid blood levels and prevent/reduce CVD. Mutations in different genes have been shown to cause monogenic dyslipidemia, including *LDLRAP1* mutations that are involved in autosomal recessive hypercholesterolemia, *ABCG5/ABCG8*, involved in sitosterolemia, or *LMF1*, which is associated with familial chylomicronemia syndrome [56]. These genes are new potential candidates for CRISPR-Cas9 genome editing. The limitation of an appropriate delivery system is nowadays averted by the use of effective specific viral vectors and nanoparticles. These newly developed vectors aim at reducing the innate and adaptive cellular responses observed in the past, including those towards particular Cas9 nucleases [142,143]. However, the permanent nature of DNA changes caused by gene editing requires a careful evaluation of this technology before it can be broadly used to treat CVD diseases. Clinical trials of novel therapeutics in humans will require constant surveillance to enable the identification of any undesirable effects and to evaluate the long-term efficacy of the treatment in terms of plasma lipid levels and markers of atherosclerosis lesions. Indeed, the choice of some therapeutic targets such as PCSK9 or ANGPLT3 seems justified by the good overall health condition of individuals with spontaneous deficiency in these genes [81,144,145,146]. The assessment of off-target effects remains the biggest challenge, since the human genome is different to tested animal models, and since inter-individual differences are also possible. A combination of in silico prediction and testing of induced pluripotent stem-cell-derived hepatocytes are among the used approaches for detection of these off-target effects.

## Figures and Tables

**Table 1:** Main animal model studies targeting genes associated with dyslipidemia.

		Species	Delivery Vehicle	Editing Method	References
PCSK9		Mouse	Adenovirus	CRISPR	[57]
		Mouse	AAV	CRISPR	[58]
		Mouse	AAV	CRISPR	[63]
		Mouse	AAV	CRISPR	[64]
		Mouse	LLN	CRISPR	[65]
		Mouse	LNP	CRISPR	[67]
		Mouse	LNP	CRISPR	[68]
		Mouse	Adenovirus	Base editor	[69]
		NHP	AAV	Meganuclease	[70]
		NHP	LNP	Base editor	[73]
		NHP	LNP	Base editor	[74]
ANGPTL3		Mouse	Adenovirus	Base editor	[85]
		Mouse	LNP	CRISPR	[88]
		Mouse	AAV	Base editor	[89]
		Mouse	AAV	Base editor	[90]
		NHP	GalNAc-LNP	Base editor	[93]
LDLR		Mouse	AAV	CRISPR	[97]
		Mouse	AAV	CRISPR	[99]
		Rabbit	*	CRISPR	[105]
		Hamster	*	CRISPR	[106]
APO	A1	Mouse	AAV	CRISPR	[113]
	B	Mouse	AAV	CRISPR	[3]
	B	Mouse	AAV	CRISPR	[63]
	C3	Hamster	*	CRISPR	[119]
	C3	Rabbit	*	CRISPR	[120]

* = microinjections in embryos.

## Data Availability

Not applicable.

## Abbreviations

AAV:	Adeno-associated virus
ABE:	Adenine base editor
ALT:	Alanine aminotransferase
ANGPTL3:	Angiopoietin like 3
Apo(a):	Apoliporotein (a)
ApoA1:	Apolipoprotein A1
ApoB:	Apolipoprotein B
ApoC3:	Apolipoprotein C3
ASGPR:	Asialoglycoprotein receptor
AST:	Aspartate aminotransferase
Cas9:	CRISPR-associated protein 9
CRISPR:	Clustered regularly interspaced short palindromic repeats
CVD:	Cardiovascular diseases
ER:	Endoplasmic reticulum
FDA:	*Food and Drug Administration*
*FH:*	Familial hypercholesterolemia
GalNac:	Multi-valent *N*-AcetylGalactosamine
gRNA:	guide RNA
HDL:	High-density lipoprotein
HMG-CoA:	β-Hydroxy β-methylglutaryl-CoA
HoFH:	Homozygous familial hypercholesterolemia
KO:	Knockout
LDL:	Low-density lipoprotein
LDLR:	Low-density lipoprotein receptor
LLN:	Lipid-like nanoparticle
LNP:	Lipid nanoparticle
Lp(a):	Lipoprotein A
NHP:	Non-human primates
PCSK9:	Proprotein convertase subtilisin/kexin type 9
PPAR-alpha:	Peroxisome proliferator-activated receptor alpha
RNP:	Ribonucleoprotein
RXR:	Retinoid X receptor
siRNA:	Small interfering RNA
TC:	Total cholesterol
TG:	Triglycerides
VLDL:	Very low density lipoprotein
WT:	Wild-type
